# SubPatCNV: approximate subspace pattern mining for mapping copy-number variations

**DOI:** 10.1186/s12859-014-0426-7

**Published:** 2015-01-16

**Authors:** Nicholas Johnson, Huanan Zhang, Gang Fang, Vipin Kumar, Rui Kuang

**Affiliations:** 1 Department of Computer Science and Engineering, University of Minnesota, Minneapolis, USA; 2 Department of Genetics and Genomic Sciences, Mount Sinai School of Medicine, New York, USA

**Keywords:** DNA copy-number variations, Approximate pattern mining, HapMap, Cancer

## Abstract

**Background:**

Many DNA copy-number variations (CNVs) are known to lead to phenotypic variations and pathogenesis. While CNVs are often only common in a small number of samples in the studied population or patient cohort, previous work has not focused on customized identification of CNV regions that only exhibit in subsets of samples with advanced data mining techniques to reliably answer questions such as “Which are all the chromosomal fragments showing nearly identical deletions or insertions in more than 30% of the individuals?”.

**Results:**

We introduce a tool for mining CNV subspace patterns, namely SubPatCNV, which is capable of identifying all aberrant CNV regions specific to arbitrary sample subsets larger than a support threshold. By design, SubPatCNV is the implementation of a variation of approximate association pattern mining algorithm under a spatial constraint on the positional CNV probe features. In benchmark test, SubPatCNV was applied to identify population specific germline CNVs from four populations of HapMap samples. In experiments on the TCGA ovarian cancer dataset, SubPatCNV discovered many large aberrant CNV events in patient subgroups, and reported regions enriched with cancer relevant genes. In both HapMap data and TCGA data, it was observed that SubPatCNV employs approximate pattern mining to more effectively identify CNV subspace patterns that are consistent within a subgroup from high-density array data.

**Conclusions:**

SubPatCNV available through http://sourceforge.net/projects/subpatcnv/is a unique scalable open-source software tool that provides the flexibility of identifying CNV regions specific to sample subgroups of different sizes from high-density CNV array data.

**Electronic supplementary material:**

The online version of this article (doi:10.1186/s12859-014-0426-7) contains supplementary material, which is available to authorized users.

## Background

DNA copy-number variations — deletion or amplification of DNA segments — are one type of structural aberrations that could lead to phenotypic variations and pathogenesis. For example, it has been previously reported that many CNVs are responsible for cellular function abnormalities in cancer [1,2]. Being able to characterize and identify these causal CNVs is essential in understanding the molecular mechanisms of cancer for developing effective treatment. Advanced high-throughput genomic technologies for array-based comparative genomic hybridization (CGH) or genotyping arrays are capable of running genome-wide studies to characterize copy-number variations. CNV data analysis is becoming increasingly important as more and more CNV data are accumulated for international effort in cancer in The Cancer Genome Atlas (TCGA) and population genetics studies in HapMap [3]. However, as an important source of genetic variations spanning a larger fraction of genome than single nucleotide polymorphisms (SNPs), CNV data was under utilized in those studies. Previous studies taking advantage of the high-throughput technology to identify driver CNVs share little agreement and, as such, the results are difficult to decipher [4,5] given the uncertainties in CNV data analysis by different computational methods, in particular, heuristic evaluations of an exponential number of combinations of regions of consecutive probe features in arbitrary subsets of samples. It is evident that advanced data mining algorithms, similar to those methods for difficult SNP analysis problems in GWAS [6], are in great needed for CNV analysis.

In this paper, we introduce a tool for mining CNV subspace patterns called SubPatCNV. The pattern mining methodology that SubPatCNV is built upon overcomes the computational demand of evaluating an exponential number of patterns. Specifically, SubPatCNV is an approximate association pattern mining algorithm [7] under a spatial constraint on the copy-number variation probe features. The approximate association pattern mining framework allows consideration of large, common CNV regions across patient subsets as well as exhaustive analysis of all potential aberrant CNV regions. SubPatCNV provides the flexibility to identify aberrant CNV regions that are specific to a subset of patients and correlates clinical variables to patient subsets for cancer subtype discovery.

To evaluate how effectively SubPatCNV can detect CNV patterns relevant to true patient subgroups as a benchmark test, SubPatCNV was applied to analyze a collection of HapMap samples of high-density arrays. In the test, SubPatCNV was capable of detecting large germline CNV patterns in which the support samples are only specific to one of the four populations in the HapMap samples. SubPatCNV was further tested on ovarian cancer CNV datasets to demonstrate its application in cancer studies. SubPatCNV reported CNV regions highly enriched with cancer-relevant genes.

## Methods

**Figure 1:**
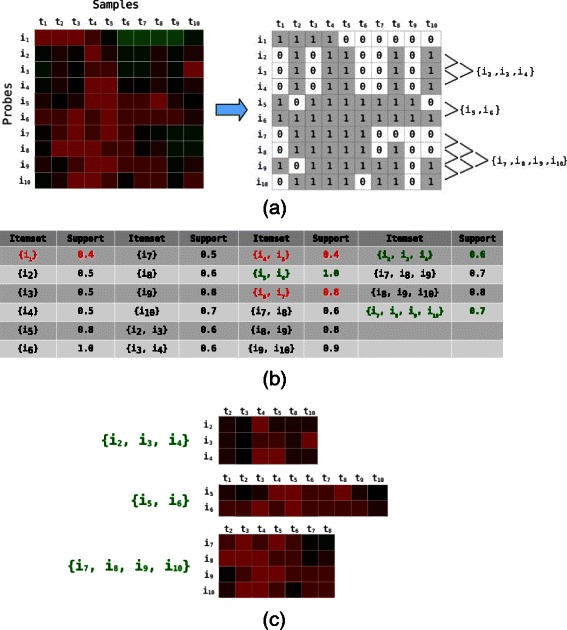
**SubPatCNV workflow on a toy dataset.** **(a)** CNV data are discretized. **(b)** The approximate pattern mining algorithm (SubPatCNV) is applied to discover frequent CNV patterns with the support threshold 0.5, error tolerant rate *ε*=0.2, and merge threshold *δ*=0.4. The itemsets in red are pruned by the support threshold or the merging criteria. The green itemsets are the maximum frequent itemsets by the criteria. Note that by convention approximate pattern mining does not allow any error tolerance for singleton itemsets and thus the itemset { *i*_1_} is pruned. **(c)** CNV subspace patterns are visualized as the original log-intensity profiles.

This section describes the methodology of SubPatCNV for data preparation, approximate pattern mining and implementation. Figure 1 outlines the workflow of SubPatCNV.

### Data preprocessing

In approximate association pattern mining, the dataset is first transformed into a *m*×*n* binary matrix where *m* is the number of items and *n* is the number of transactions. For array-based CNV data, items are the probes and transactions are the patient samples and each entry is a log intensity ratio between the sample and a reference. In normal DNA regions, the log intensity ratio close to 0 indicates that the sample has the same copy number as the reference. A copy number gain or loss is indicated by a 1 if the absolute value of the log intensity is larger than a threshold *τ* and otherwise 0 as shown in Figure 1a. The threshold *τ* defines the range of common CNV values and any values outside of this range are candidate CNVs of interest. Note that copy number gain and loss are analyzed separately by pattern mining. The threshold *τ* can be empirically set to define a predetermined sparsity level in the dataset, and its value impacts the number of patterns discovered as shown in the experiments.

### Approximate frequent patterns

Consider a binary dataset that consists of transactions *T*={*t*_1_,*t*_2_,…,*t*_*n*_} and items *I*={*i*_1_,*i*_2_,…,*i*_*m*_}. Each *t*_*i*_ is a transaction that contains a subset of items from *I*. If we represent the dataset as a matrix *D* and let rows correspond to items and columns correspond to transactions then *D* will be a *m*×*n* binary matrix where *D*_*j*,*k*_=1 if *i*_*j*_∈*t*_*k*_, and 0 otherwise. For any itemset *I* ^′^⊂*I*, its itemset support is defined as the number of transactions that contain *I* ^′^, i.e., |*T* ^′^:*I* ^′^⊂*t* ^′^,∀*t* ^′^∈*T* ^′^ and *I* ^′^⊄*t* ^′′^,∀*t* ^′′^∈*T*−*T* ^′^|. An itemset is considered a frequent itemset if its support is greater than a user-specified threshold *s* *u* *p*_*min*_.

#### Error tolerant itemsets (ETIs)

To account for noise in CNV data, SubPatCNV is based on a variation of the Dense Itemsets algorithm [8] developed for discovering approximate frequent patterns that allow a specified percentage of items to be missing from the itemset which are often called error-tolerant itemsets (ETIs). Two kinds of ETIs were defined in [9]: strong and weak.

An ETI *I* ^′^ is said to be weak with tolerance *ε* if ∃*T* ^′^⊂*T* such that |*T* ^′^|≥*s* *u* *p*_*min*_ and $\frac {\sum _{i \in I^{\prime }}\sum _{t \in T^{\prime }} D_{i,t}}{|I^{\prime }|*|T^{\prime }|} \geq 1-\epsilon $. Such a definition does not specify the errors in each row and column and thus it allows for rows or columns to be completely empty or consisting of all 0’s. An ETI *I* ^′^ is said to be strong with tolerance *ε* if ∃*T* ^′^⊂*T* such that |*T* ^′^|≥*s* *u* *p*_*min*_ and $\forall t \in T^{\prime }: \frac {\sum _{i \in I^{\prime }} D_{i,t}}{|I^{\prime }|} \geq 1-\epsilon $. A strong ETI adds the constraint that each transaction must contain a certain fraction of items in order to avoid spurious transactions that contain all 0’s. However, strong ETIs often discover very few useful associations due to their strong requirements. ETI algorithms have been developed to find a balance between spurious item requirements and useful frequent itemset discovery such as Dense itemsets [8].

The Dense Itemsets algorithm overcomes spurious items in the itemset by adding a recursive constraint into the weak ETI definition. The itemsets discovered by the Dense Itemsets algorithm are called *recursive weak error tolerant itemsets*. In this algorithm an itemset *I* ^′^ is a recursive weak ETI if *I* ^′^ and all subsets of *I* ^′^ are weak ETIs. This algorithm is useful since it adds a natural pruning heuristic in that if an itemset is not a recursive weak ETI then no superset can be a recursive weak ETI and thus can be pruned in the combinatorial search.

### Subspace patterns in copy-number variation discovery

SubPatCNV discovers recursive weak ETIs of consecutive probe items with two specific changes to the Dense Itemsets algorithm catered to CNV data. First, we only consider sets of consecutive probes as candidate frequent itemsets. Second, we limit itemset merging by requiring the two itemsets to be similar in support to one another. Below we outline the two changes.

Traditional approximate association pattern mining algorithms, such as Apriori [10] and Dense Itemsets [8], consider all combinations of items to be potentially interesting given that the items do not have meaningful orders associated with them. Directly applying existing approximate association pattern mining algorithms to CNV datasets with hundreds of thousands of features would not be computationally feasible.

To address this computational challenge, SubPatCNV implements a CNV data specific pruning to take advantage of the positional correlations among consecutive probes in order to scale to large CNV datasets. Probes that are nearby to one another tend to be involved in the same CNV event while probes that are far away do not. As such, the pruning strategy takes this into account by only considering sets of items (probes) that are continuous. SubPatCNV first calculates the support of each individual probe and if the support is <*s* *u* *p*_*min*_ then the probe is not considered a frequent itemset and is discarded. This is illustrated in Figure 1a and 1b. In this example, *s* *u* *p*_*min*_=0.5 and probe *i*_1_ has a support of 0.4<*s* *u* *p*_*min*_, and thus this probe is discarded. SubPatCNV then proceeds in rounds by considering each individual frequent itemset from the previous round and tries to merge them together. Each itemset will be merged if two criterion are passed. The first criterion utilizes the positional dependence between probes by only allowing frequent itemsets to be merged with their neighbor frequent itemsets along the chromosome. If the two frequent itemsets only differ in the first probe and the last probe, which means that the itemsets contain consecutive probes, they pass this positional dependence requirement.

The second criterion is that the two frequent itemsets to be merged need to share enough overlapping support with one another. A user-defined parameter *δ* is introduced to represent the dissimilarity between two itemsets to be merged as the percentage of non-overlapping transactions. The two itemsets are required to share exactly the same support when *δ*=0.0 and have no support requirement when *δ*=1.0. For example in Figure 1b with *δ*=0.4, the itemset { *i*_6_,*i*_7_} is discarded with 0.8>*s* *u* *p*_*min*_ because the itemsets { *i*_6_} and { *i*_7_} have support, 1.0 and 0.5 respectively, that differ too much. This requirement is useful because a frequent itemset might have a high enough support that it gets merged repeatedly with other itemsets with much lower support. These itemsets may have very different signatures and merging them together would otherwise fail to capture both unique itemsets individually.

If both criterion are passed then the two itemsets will be merged together into a larger candidate frequent itemset; otherwise the itemsets to be merged will stay as individual frequent itemsets. Finally, the candidate frequent itemsets will be subjected to the recursive weak ETI criterion and if an itemset passes this criterion it is deemed a frequent itemset. Otherwise, the merged frequent itemset is discarded and the individual frequent itemsets are kept.

Figure 1a visualizes the frequent itemset merges alongside the binary matrix. Figure 1b shows the support values of each candidate frequent itemset and those in red are discarded for failing to satisfy the criteria or have a support value less than *s* *u* *p*_*min*_. The frequent itemsets in green are those frequent itemsets identified by SubPatCNV as frequent CNV regions of interest, which are shown as bi-clusters in Figure 1c.

### Usage

We developed and released an open-source toolbox that implements our SubPatCNV algorithm through sourceforge [11]. We provide detailed instructions on how to use the tool and examples on a real dataset. The tool was built to be easy to use with little to no preprocessing of the data. The main tool that implements the SubPatCNV algorithm is called via the command line, for example, ./subpatcnv chr_data sup eps delta > output where the first argument chr_data is the binarized data for a specific chromosome, the second argument sup is the desired support level, the third argument eps is the weak ETI tolerance, and the fourth argument delta is the merging threshold. The output can be captured in a plain text file where each row is a pattern that specifies the sample indices that support the pattern.

Additionally, we provide shell scripts to set up the file structure required by the tool and automatically run full genome experiments for varying values of the arguments. We also provide MATLAB scripts to support result analysis and visualization.

### Related work

In the literature, relatively few tools were specifically developed to identify common CNV regions across samples. GISTIC [12,13] and its variation JISTIC [14] are two tools designed to detect causal CNVs of tumor genesis from a patient cohort. GISTIC [12,13] takes a statistical approach by calculating a (probe-)positional summary statistic (G-score) for the positional CNVs across all patients in the dataset. Using this G-score, GISTIC performs permutation tests and assigns each position a q-value to evaluate the significance of the CNV in relation to background rates. Only those positions with a lower q-value than a user-defined threshold value are retained. Finally, a “peel-off” heuristic procedure iteratively selects CNV regions from the retained positions with the greatest frequency and amplitude within each continuous region of significant CNVs. The selected CNV regions are then zeroed out in all samples that contribute, and the q-values are recalculated. The iterative process repeats to grow the positions into “peak” regions. JISTIC [14] improves upon GISTIC by introducing a variation of the “peel-off” search heuristic called “limited peel-off”. JISTIC’s motivation is that it is expected that the G-score of each probe position can be decomposed into two parts: the first represents the peak to be peeled off and the second is the contributions independent to the peak. Thus, only the sample contributions that make up the first part are subtracted. In this way, JISTIC peels off the G-score portions that contribute to the current peak region while leaving the other portions that contribute to other secondary peaks.

GISTIC and JISTIC identify frequent CNV regions that might contain loci identified from different patient subgroups since calculations of G-scores do not utilize the positional dependence between probes. Those probes in the same peak might only be present in different groups of patient samples. It is reasonable to report those regions because the adjacent loci might suggest the same functional abnormality such as disruption of the function of the same gene by different somatic CNVs. However, in the case of detecting inherited germline CNVs in population genetics, the assumption is less reliable. Moreover, CNV data are often noisy and high-dimensional. Advanced computational methods are needed to explicitly handle the noise in microarray data and to evaluate all combinations of samples and probes as patterns.

## Results

Since there is no ground truth in available large-scale CNV datasets, directly benchmarking the performance of frequent CNV discovery is not possible. Instead, we benchmarked the performance of frequent CNV discovery by SubPatCNV on HapMap data [3] by utilizing the population information as a measure of the quality of the discovered frequent CNV patterns. We expect that frequent patterns which are true germline CNV patterns in some population(s) will show population preferences. To demonstrate SubPatCNV’s applicability to cancer CNV analysis, we also performed experiments on the TCGA ovarian cancer dataset.

### HapMap data preparation

The affymetrix 500k HapMap data were downloaded from the HapMap data repository [15]. The data include 30 mother-father-adult child trios from the Yoruba in Ibadan, Nigeria (YRI); 30 trios of northern and western European ancestry living in Utah from the Centre d’Etude du Polymorphisme Humain collection (CEU); 45 unrelated Han Chinese individuals in Beijing, China (CHB); and 45 unrelated Japanese individuals in Tokyo, Japan (JPT). We grouped the samples into 3 populations (YRI, CEU, CHB+JPT) with 90 samples in each. We used PennCNV-Affy protocol from PennCNV [16] to convert raw CEL files into Log R Ratio (LRR) signal intensity files. The comparison focuses on larger patterns of at least size 2 since it is easy and straightforward to detect frequent single-probe CNVs. In this section, we use population specificity on patterns to demonstrate the performance of SubPatCNV and JISTIC. In the classification test, we removed 44 samples with very large variations to retain a subset of data of high quality (see PCA analysis in Additional file 1: Figure S2). This subset contains 226 samples with 75 in CEU, 79 in CHB+JPT, and 72 in YRI.

### Hyper-parameter selection

Additional file 1: Figure S1a shows the distribution of the probe intensitie-ratios in the Hapmap data. We set a threshold *τ* as 0.192 to let 10% data to be CNV on the steep slope of the curve. There are three SubPatCNV hyper-parameters to tune - *s* *u* *p*_*min*_: the sample support for CNV patterns; *ε*: the error tolerance of ETI; and *δ*: non-overlapping between two merged itemsets.

**Figure 2:**
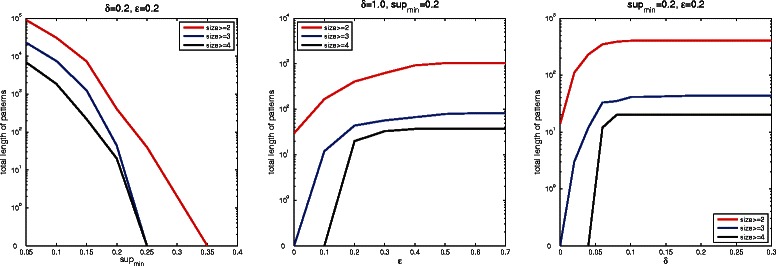
**Parameter selection on Hapmap data.** The total length of frequent CNV patterns detected under different choices of *s* *u* *p*_*min*_, *ε* and *δ* parameters. Each plot shows the total number of probe features in the frequent patterns of size at least 2, 3 and 4.

To show the effect of each parameter, we vary one parameter and fix the other two. In Figure 2, the left plot shows the higher support threshold used the fewer patterns detected by SubPatCNV. When support is larger than 35%, no frequent CNV is detected. This observation might relate to the equal proportions of the three groups in the data. The middle plot shows that larger *ε* allows more errors and thus larger patterns to be discovered. The right plot shows that intuitively, large *δ* encourages detection of larger patterns since more patterns are allowed to be merged. However, the relation stops when *δ* is above 0.15. The reason is because amplification and deletion patterns are relatively sparse (10% among all the probes). Therefore, the support is weakened or gone in merged patterns as *δ* increases as, after a certain point, there are no neighbor patterns available to merge.

### Population-specific patterns

To assess the quality of detected large frequent CNV patterns, we measured the population specificity of the patterns. SubPatCNV was used to generate patterns of support threshold *s* *u* *p*_*min*_= 10% or 20% and error tolerance *ε*= 0.2 or 0.4. For each pattern of size at least two, samples are divided into the support group and the non-support group by the dense itemsets algorithm. Based on the two groups and the populations, *p*_*v* *a* *l* *u* *e* *s* are calculated with *χ* ^2^-test for each pattern. In comparison, for each non-singleton pattern of JISTIC, the support and non-support groups were identified and then a *p*_*v* *a* *l* *u* *e* was calculated in the same way.

**Figure 3:**
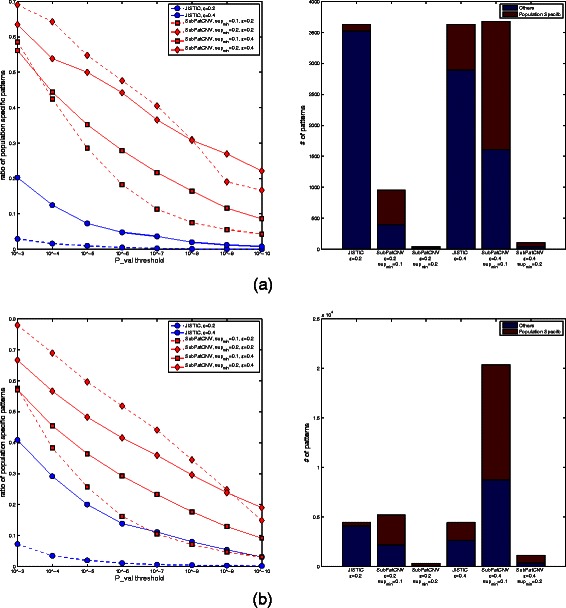
**Comparison of population specific patterns on Hapmap data.** In **(a)** and **(b)**, the left plots show the ratio of population specific patterns detected by SubPatCNV and JISTIC under different significance levels in *χ* ^2^-test of each pattern against the populations. SubPatCNV was tested under several different combinations of *ε* and sup*min*. The right histograms show the number of population specific and non-population-specific patterns detected by the two methods with *χ* ^2^-test *p*_*v* *a* *l*<10^−3^.

Figure 3 reports the detected patterns by population specificity. The left plots show the percentage of population-specific patterns detected by the methods under the different combinations of *s* *u* *p*_*min*_ and *ε*. SubPatCNV detected higher ratio of population specific patterns in all the combinations of the parameters. With the least stringent cutoff on *p*_*v* *a* *l* *u* *e* at 10^−3^, 55*%*−70*%* of the patterns detected by SubPatCNV are population-specific. The lower ratio of population-specific patterns detected by JISTIC could be due to either the incoherence within the CNV regions or the noise in the array data. To further explain why SubPatCNV detected more population-specific patterns, several examples of patterns are shown and discussed in the next section.

**Figure 4:**
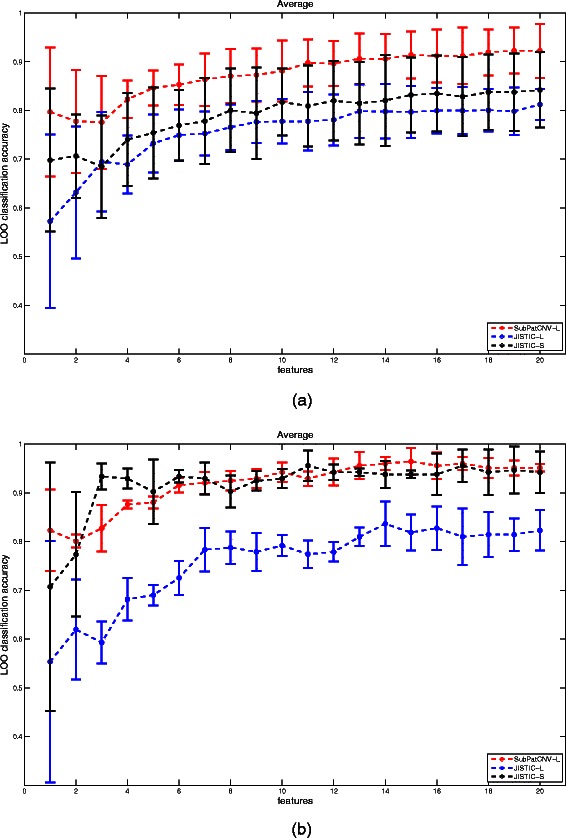
**Discriminative frequent CNV patterns of Hapmap data.** The plot shows leave-one-out classification accuracy on a portion of the 3 populations using frequent CNV patterns selected by remaining individuals. **(a)** Leave-one-out accuracy on 90% of the samples using patterns selected by 10% of the samples. **(b)** Leave-one-out accuracy on one third of the samples using patterns selected by two thirds of the samples. The x-axis shows the number of patterns used for classification where the patterns are first ranked based on their population specificity. SubPatCNV-L stands for SubPatCNV patterns with size at least 2; JISTIC-L and JISTIC-S stands for JISTIC patterns with size at least 2 or all patterns including singletons respectively.

To test whether the large frequent CNV patterns fully describe genetic characteristics of the populations, we performed a cross-validation with forward selection to choose patterns based on the *p*_*v* *a* *l* *u* *e* *s* on a set of samples and then applied leave-one-out classification with support vector machine on the remaining samples. Note that patterns with more than one probes are averaged as one feature in classification. The results are shown in Figure 4. The large patterns selected by SubPatCNV are compared with the patterns selected by JISTIC with only the large patterns (JISTIC-L) or all the patterns including the singletons (JISTIC-S) in forward selection. When only 10*%* of data are used to rank the patterns in Figure 4a, SubPatCNV clearly identified patterns that are more predictive of the populations than JISTIC. *p*_*v* *a* *l* *u* *e*s by t-test in most of the cases with different number patterns used for classification are significant by cutoff 0.05. When more data is available for ranking the patterns in Figure 4b, the large patterns selected by SubPatCNV are more predictive than the large ones selected by JISTIC and comparable to all the patterns including singletons. Note that the large patterns by SubPatCNV show smaller variance compared with the JISTIC singletons. The observations suggest that when less data are labeled for pattern selection for classification, large patterns are usually more consistent between the set used for pattern selection and the set used for classification test since large patterns are often more population specific compared with singletons. While some singletons might be very predictive, there are also more non-population specific features selected with a small label set. When more data is labeled for pattern selection, there are less false positive singletons selected. However, since there are many more singletons compared with larger patterns, higher variance is also expect even if the average performance is similar. To better illustrate how the selected patterns are predictive of the populations, Additional file 1: Figure S3 shows a few cross-validation examples. It is clear that the larger patterns selected by SubPatCNV show high consistency in the pattern selection set and the classification set.

**Figure 5:**
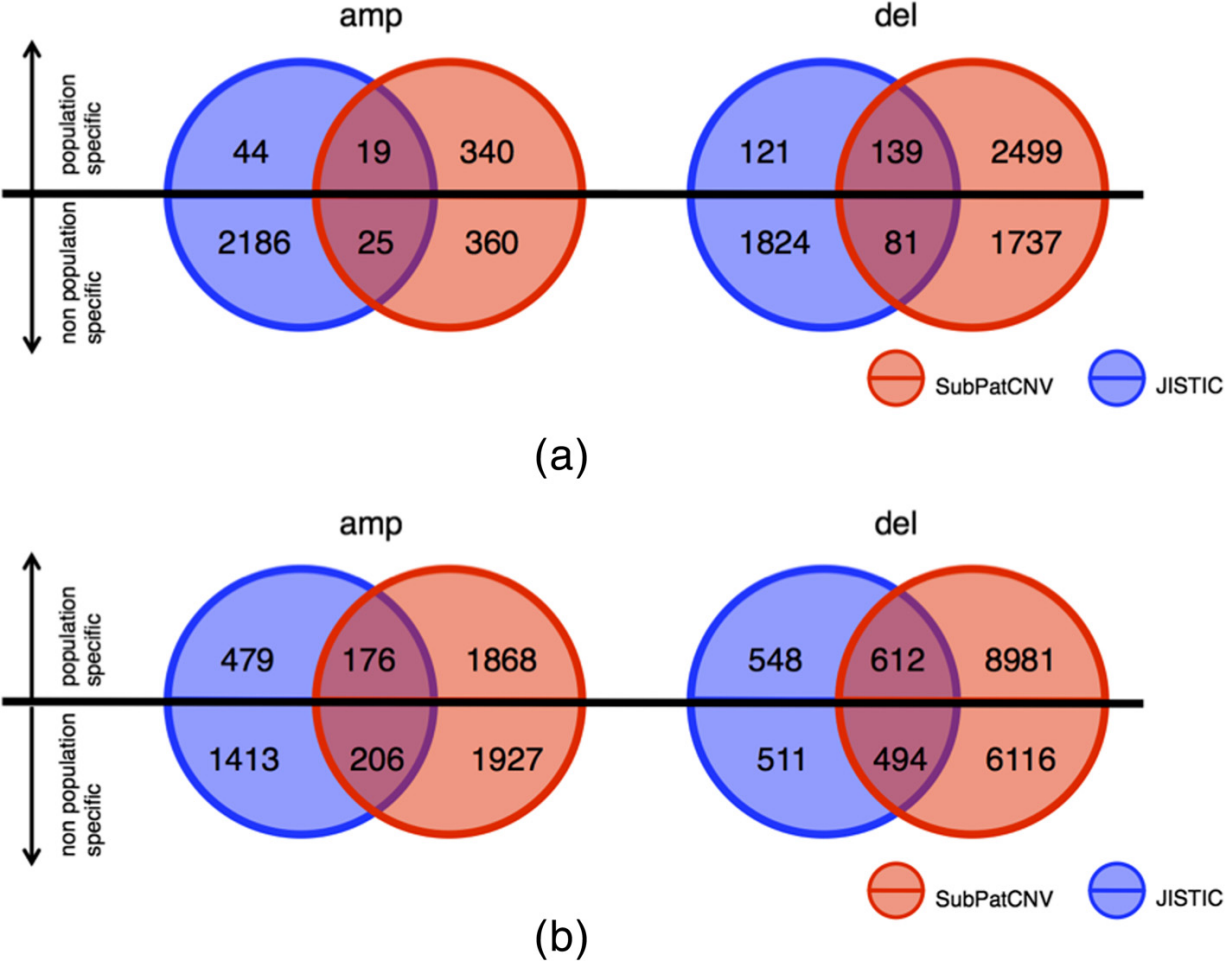
**Overlapping of CNV patterns of size 2 or larger detected by SubPatCNV and JISTIC on Hapmap data.** To show more comparable numbers of patterns detected by the two methods, *δ*=0.2 and *s* *u* *p*_*min*_=0.1 were chosen for SubPatCNV. The Venn diagrams **(a)** and **(b)** shows amplification and deletion patterns under *ε*=0.2 and 0.4 respectively. Each circle is further divided by a horizontal line with the upper half representing population-specific patterns (*p*_*v* *a* *l*<10^−3^) and the lower half representing the non-population-specific patterns.

Figure 5 shows the overlap of frequent CNV patterns of size at least 2 predicted by SubPatCNV and JISTIC. Among all the overlapping patterns, a much higher ratio of population-specific patterns detected by JISTIC are also detected by SubPatCNV. The comparison shows that SubPatCNV indeed capture many more population-specific patterns detected by JISTIC.

### Illustrations of frequent CNV patterns

To further establish the role of approximate pattern mining in detecting more population-specific patterns, we illustrate examples of patterns detected by JISTIC and SubPatCNV to discuss the potential weaknesses and the strengths of the two methods.

**Figure 6:**
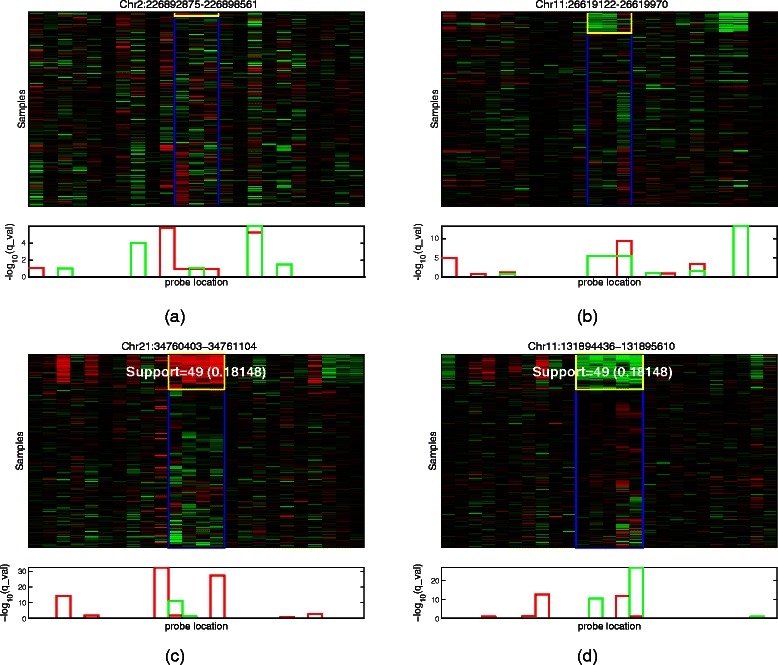
**Coherent and incoherent CNV patterns on Hapmap data.** Patterns are annotated as the regions between two blue vertical lines shown in the heatmap of the original log-intensity-ratios. The plots below the heatmaps show the *q*_*v* *a* *l* *u* *e* of each probe calculated with JISITC. **(a)** and **(b)** are incoherent patterns and **(c)** and **(d)** are coherent patterns detected by SubPatCNVs. The yellow rectangles show the supports reported by JISTIC in **(a)** and **(b)** and by SubPatCNV in **(c)** and **(d)**.

JISTIC does not explicitly use positional dependence in pattern discovery, and thus might detect patterns of inconsistent intensity profiles. Figures 6a and 6b show two such patterns. By the visualization, it is clear that the samples support each probe in the whole pattern are inconsistent. For example, in Figure 6b, for the size-3 deletion pattern, the support samples reported by JISTIC only show consistency on the first two probes but miss the third probe. Therefore, this pattern is not a peak reporting a single CNV event. SubPatCNV builds on approximate association pattern mining algorithm to accommodate noise in the pattern. Figure 6c and 6d shows two patterns detected by SubPatCNV with at least 15% support. In the examples, JISTIC reported fragmentary CNV regions due to the noise in the data while SubPatCNV accurately captured complete probe set as a single pattern. Additional file 1: Figure S9 shows the maximum support under different fraction of probes in a pattern (region). The two JISTIC patterns have a drastically lower support when larger fractions (e.g. >50%) of probes are considered, which indicates that very few samples contain coherent CNVs in all the probes. The two SubPatCNV have relatively flat trend indicating higher coherence acrvoss all the probes. For example, the JISTIC amplification pattern has a 50% support when one probe is considered and a much lower 7% support when two probes are considered. More such patterns are shown in Additional file 1: Figure S4 and S5.

### Results on TCGA ovarian cancer data

The TCGA dataset was downloaded from TCGA data-portal [17]. SNP level 2 tangent data, generated from Affymetrix Genome-Wide Human SNP Array 6.0 platform. There are 1,737,682 probe measurements from chromosome 1 to 22. Patient samples were classified into two groups with survival time less than 1 year as positive samples and longer than 5 years as negative samples. In total, 124 patient samples (46 positive and 78 negative) were included in the experiment. Additional file 1: Figure S1b shows the data distribution. We set *τ* to be 0.6227 to allow a 20% density of the data to be CNV signals since more CNV events are expected in cancer data. SubPatCNV detected 388,214 patterns, out of which 23.4% were of size at least two. JISTIC did not scale to this high density CNV dataset and terminated with errors. By running JISTIC on each individual chromosome by modifying the code, we were able to detect 287,908 peak results from all the chromosomes except chromosome 14 with JISTIC package. Only about 4.1% of the patterns detected by JISTIC were of size at least two, and most of the patterns are single probe CNVs.

**Figure 7:**
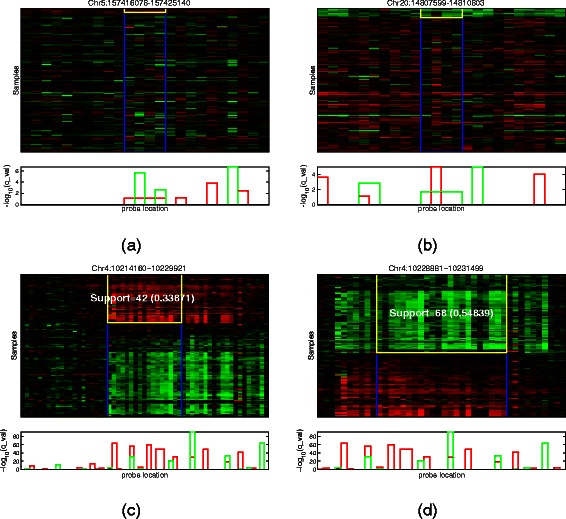
**Coherent and incoherent CNV patterns on ovarian cancer data.** Patterns are annotated as the regions between two blue vertical lines shown in the heatmap of the original log-intensity-ratios. The plots below the heatmaps show the *q*_*v* *a* *l* *u* *e* of each probe calculated by JISITC. **(a)** and **(b)** are incoherent patterns and **(c)** and **(d)** are coherent patterns detected by SubPatCNVs. The yellow rectangles show the supports reported by JISTIC in **(a)** and **(b)** and by SubPatCNV in **(c)** and **(d)**.

Figure 7a and 7b show inconsistent CNV regions reported by JISTIC. Similarly, the samples that support each probe in one region are not consistent. Figure 7c and 7d show two large-size patterns detected only by SubPatCNV. Clear consistent support can be easily identified. In these patterns, JISTIC reported fragmented CNVs. For example, in Figure 7c, SubPatCNV clearly identified a large amplification region in about one-third of the patients while JISTIC missed the region by reporting five fragmented smaller regions. The plots of support against the probe ratios in Additional file 1: Figure S10 similarly show that the JISTIC patterns have lower support while SubPatCNV patterns have relatively stable supports as the fraction of probes gets larger. More such patterns are shown in Additional file 1: Figure S7and S8.

We also correlated the discovered group-specific patterns with ovarian cancer genes. We found 14 patterns with support at least 20% and with a *p*_*v* *a* *l* *u* *e* less than 10^−4^ between the two groups. Table 1 shows the regions of the CNV patterns and the overlapping genes, four of which are known ovarian cancer genes. Low BMPR2 expression [18] and down-regulated UNC5C [19] are associated with epithelial ovarian cancer. DLEU1 was related with increased frequency of loss in the chemoresistant [20] and RNASEH2B had reduced expression involved in inhibition of gene transcription and expression [21] in ovarian cancer. The associated GO annotations of the four genes are shown in Additional file 1: Figure S11. Collectively, the evidences support that SubPatCNV is capable of detecting larger CNV events in subset of patients from high-density cancer CNVdatasets.

**Table 1:** **Group-specific CNV patterns detected by SubPatCNV on TCGA ovarian cancer data**

**Chromosome**	**BP Start**	**BP Stop**	***p*** **_value**	**Gene**	**Type**
2	203,309,381	203,309,495	9.9e-05	BMPR2	amp
5	18,404,830	18,405,744	5.7e-06		amp
7	153,041,965	153,042,465	6.6e-06		amp
4	96,394,794	96,395,108	9.1e-05	UNC5C	del
4	104,496,565	104,496,585	4.6e-05		del
4	123,047,859	123,049,424	1.1e-05		del
4	10,278,3351	102,787,549	9.1e-05	BANK1	del
13	29,955,805	29,955,844	7.9e-05	MTUS2	del
13	37,798,703	37,799,335	7.4e-05		del
13	50,741,528	50,741,888	3.7e-05	DLEU1	del
13	51,515,940	51,516,056	7.9e-05	RNASEH2B	del
13	110,545,737	110,546,191	7.9e-05		del
21	17,086,255	17,086,366	5.5e-05		del
21	24,418,912	24,419,862	9.1e-05		del

## Discussions and conclusions

In this paper, we motivate and introduce a new copy-number variation discovery software tool called SubPatCNV. This tool is based on different fundamentals compared with other existing methods since it is built on approximate association pattern mining techniques and takes advantage of the inherit correlation between log-intensity values of nearby probes to improve the pruning strategy in order to search through very large CNV datasets. SubPatCNV are excel in identifying all CNV events as a coherent regions specific to sample subgroups. In Additional file 1: Table S1, we report the overall density of CNV patterns in the chromosomes. The densities varies by the cutoff for binarization and support but are comparable to the 0.5% density of the HapMap Consortium CNV calls (http://hapmap.ncbi.nlm.nih.gov/downloads/cnv_data/). For example, when the binarization is 1% density and the support is 0.1, the overall density of the CNV patterns is also 0.5%. The comparison shows that the CNV discovery trends with segmentation of individual samples or pattern discovery of subgroups are consistent with the HapMap data.

Note that GISTIC and JISTIC were primarily developed to identify recurrent CNVs, which are not necessarily coherent patterns. Thus, the primarily focus of the comparison with JISTIC in the experiments on the HapMap data is to show that SubPatCNV is a more appropriate tool to identify CNVs in population stratification. The same conclusion also applies to cancer CNV data if the primary goal is to detect the same patterns. However, in general, SubPatCNV probably will not perform better than GISTIC or JISTIC to identify recurrent cancer CNVs regardless of whether the CNVs in a region co-occur. In addition, another limitation of SubPatCNV is absence of the overall significance of the patterns. Evaluation of the statistical significance of pattern mining requires advanced statistical approaches such as swap permutations, multiple testing correction and holdout set evaluation [22,23], which are often not directly applicable in large real datasets with approximate patterns. Other advanced methods such as large-margin-based clustering [24] can detect subsets with globally similar CNV profiles without focusing on detecting local patterns. Thus, SubPatCNV is a useful unique tool that is highly supplementary to the existing tools.

Recently, the new generations of DNA sequencing technology also provide short read data for CNV detection. Previous work demonstrated that when a reference data is available, CNV detection from the short reads is also reliable. In the future, we will extend SubPatCNV to include a second module to handle short read data for the same analysis.

## Availability and requirements

**Project name:** SubPatCNV **Project website:** http://sourceforge.net/projects/subpatcnv/ **Operating system(s):** Platform independent **Programming language:** C++ / MATLAB **Other requirements:** None **License:** Free **Any restrictions to use by non-academics:** None

## Additional file

Additional file 1
**contains additional experimental results on the HapMap data and the TCGA data.**

## Abbreviations

CNV: Copy number variations
ETI: Error tolerant itemset
arrayCGH: Array-based comparative genomic hybridization
SubPatCNV: Subspace patterns for copy-number variation discovery
TCGA: The cancer genome atlas
GISTIC: Genomic identification of significant targets in cancer
