# Supplementary material for: SubPatCNV: approximate subspace pattern mining for mapping copy-number variations
Source: BMC Bioinformatics. 2015 Jan 16;16:16. doi: 10.1186/s12859-014-0426-7 (PMC4305219; doi:10.1186/s12859-014-0426-7)
Supplement: Additional file 1 — contains additional experimental results on the HapMap data and the TCGA data. [file 12859_2014_426_MOESM1_ESM.pdf]

SubPatCNV: Approximate Subspace Pattern Mining for Mapping Copy-Number Variations

Nicholas Johnson[1], Huanan Zhang[1], Gang Fang[1,2], Vipin Kumar[1], Rui Kuang[1,*]

1 Department of Computer Science and Engineering, University of Minnesota Twin Cities
2 Department of Genetics and Genomic Sciences, Mount Sinai School of Medicine, New York, New York
∗ Correspondance: kuang@cs.umn.edu

Supplementary Figures and Tables

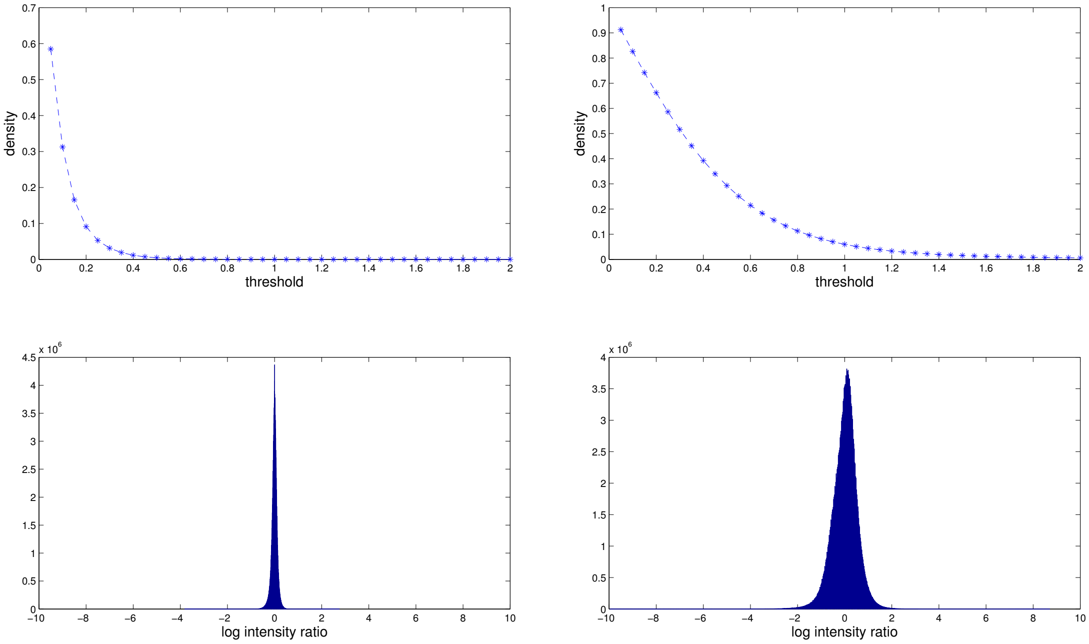

(a) HapMap Data

(b) Ovarian Cancer Data

Figure S1: **Distribution of log-intensity-ratios in the Hapmap and ovarian cancer datasets.** (a) Hapmap data. (b) TCGA ovarian cancer data. The upper figures show the density of the data above different thresholds. The lower figures show histograms of log-intensity-ratios in the two datasets. Based on the observation that CNVs events are more frequent in cancer genomes, the thresholds at the steep slopes were chosen to allow 10% and 20% density in the Hapmap and ovarian cancer datasets, respectively.

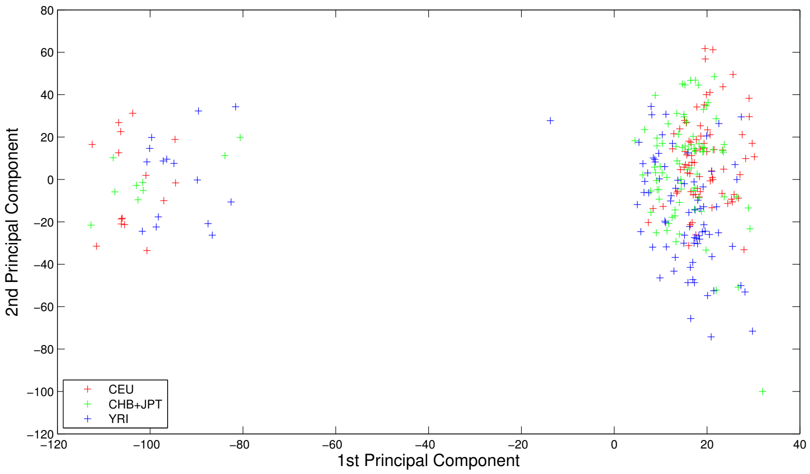

Figure S2: **Plot of the first 2 principle components of Hapmap samples.** There are clearly two clusters of individuals of 44 and 226 in each respectively with no population specificity. To avoid potential experimental noise, only the 226 individuals in the larger cluster were considered for classification test.

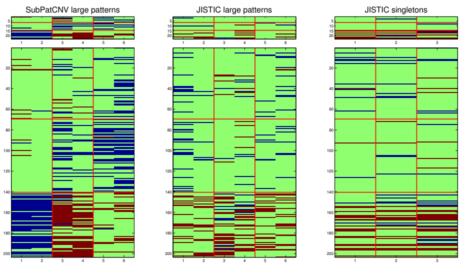

(a) cross validation 2

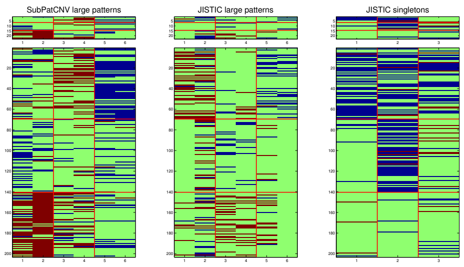

(b) cross validation 4

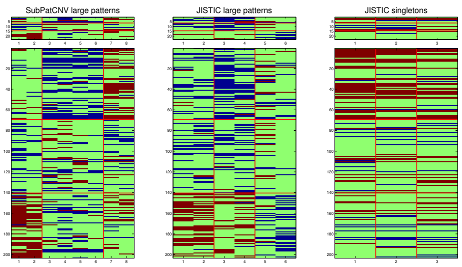

(c) cross validation 5

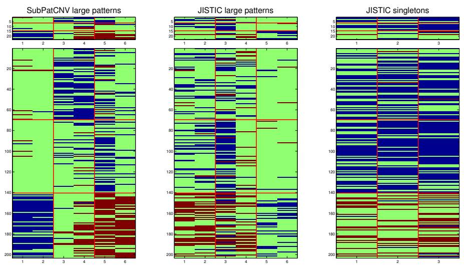

(d) cross validation 8

Figure S3: **Cross validation examples of population specific patterns on Hapmap data.** Examples shows top 3 patterns detected by SubPatCNV(large patterns), JISTIC(large patterns) and JISTIC(singletons). In each fold example, top figure stands for training and bottom figure stands for test data.

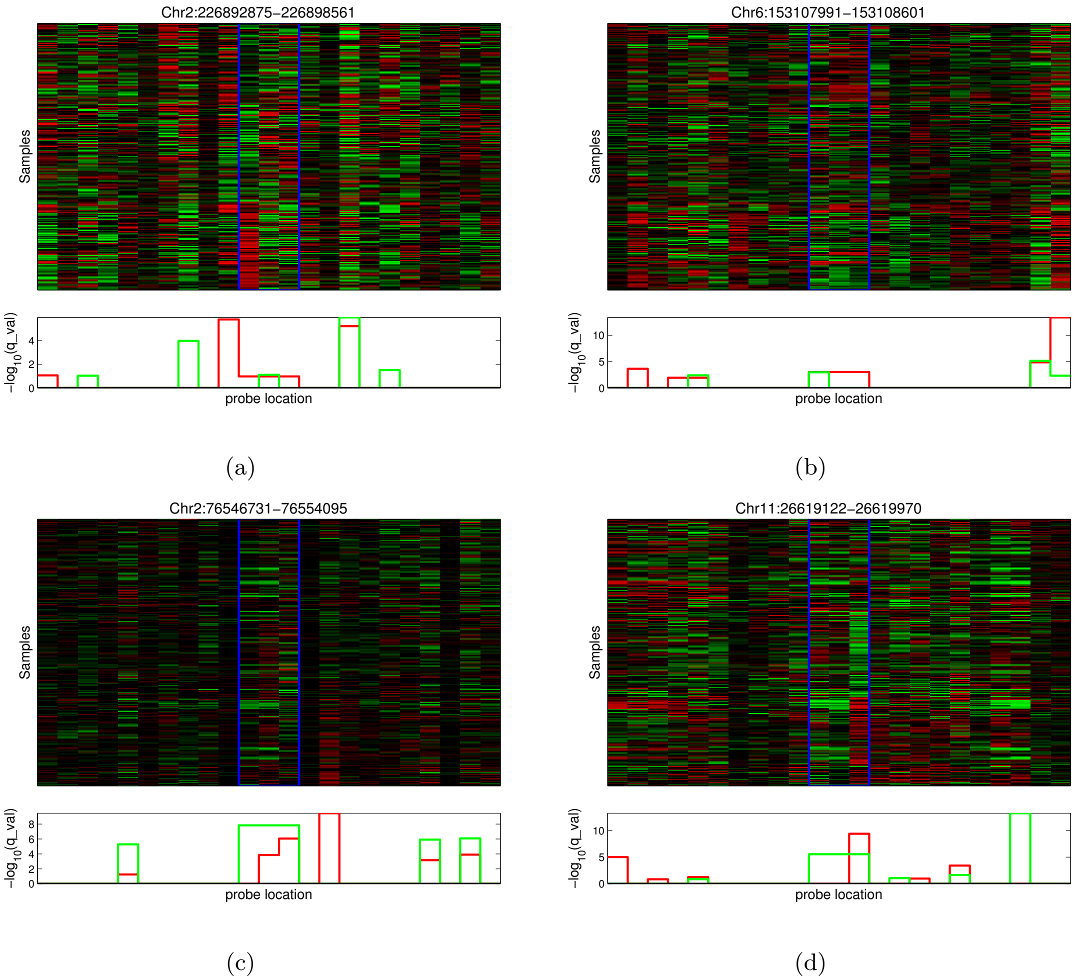

Figure S4: **Incoherent patterns detected on Hapmap data.** In the heatmaps, patterns are annotated as regions between two blue vertical lines shown in original log-intensity in HapMap. The plots below the heatmaps shows *q_value* of each probe calculated by JISITC. (a) and (b) are amplification patterns and (c) and (d) deletion patterns.

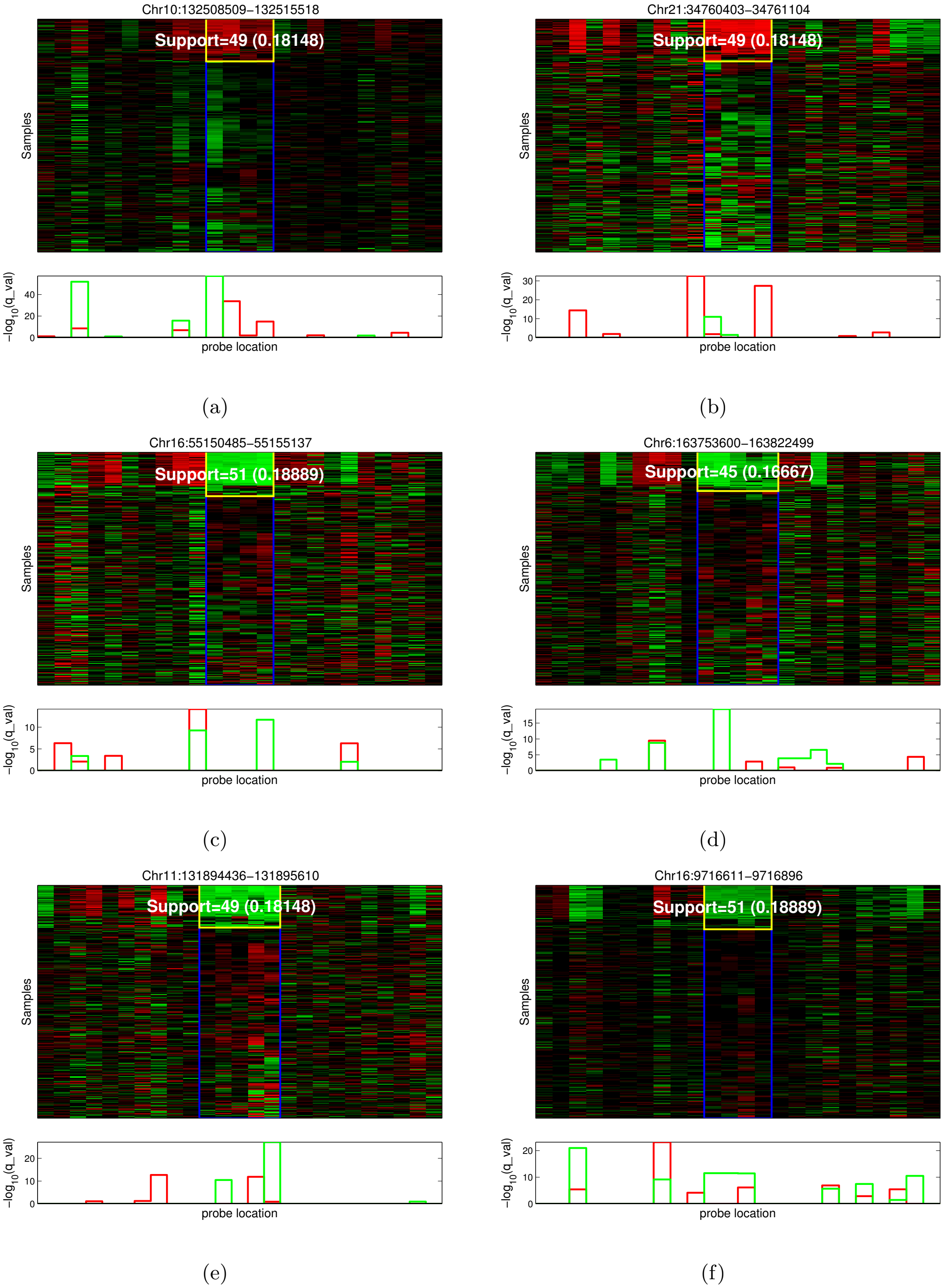

Figure S5: **Coherent patterns detected by SubPatCNV on Hapmap data.** The yellow rectangles mark the subset of samples that support the patterns. The plots below the heatmap shows *q_value* of each probe calculated by JISITC.

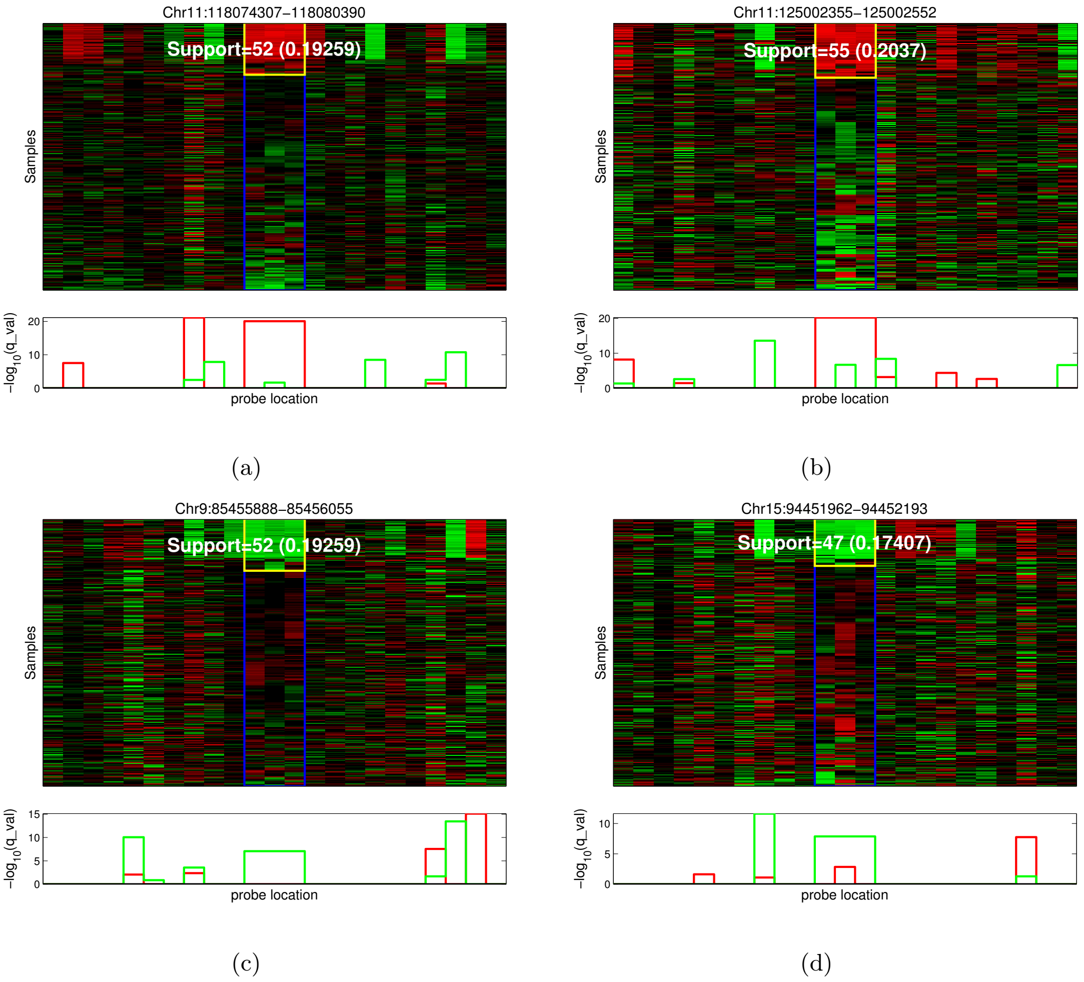

Figure S6: **Coherent patterns detected by both JISTIC and SubPatCNV on Hapmap.** The yellow rectangles display the subset of samples that support the patterns. The plots below the heatmaps show *q_value* of each probe calculated by JISITC. SubPatCNV and JISTIC detected coherent and meaningful patterns in these example.

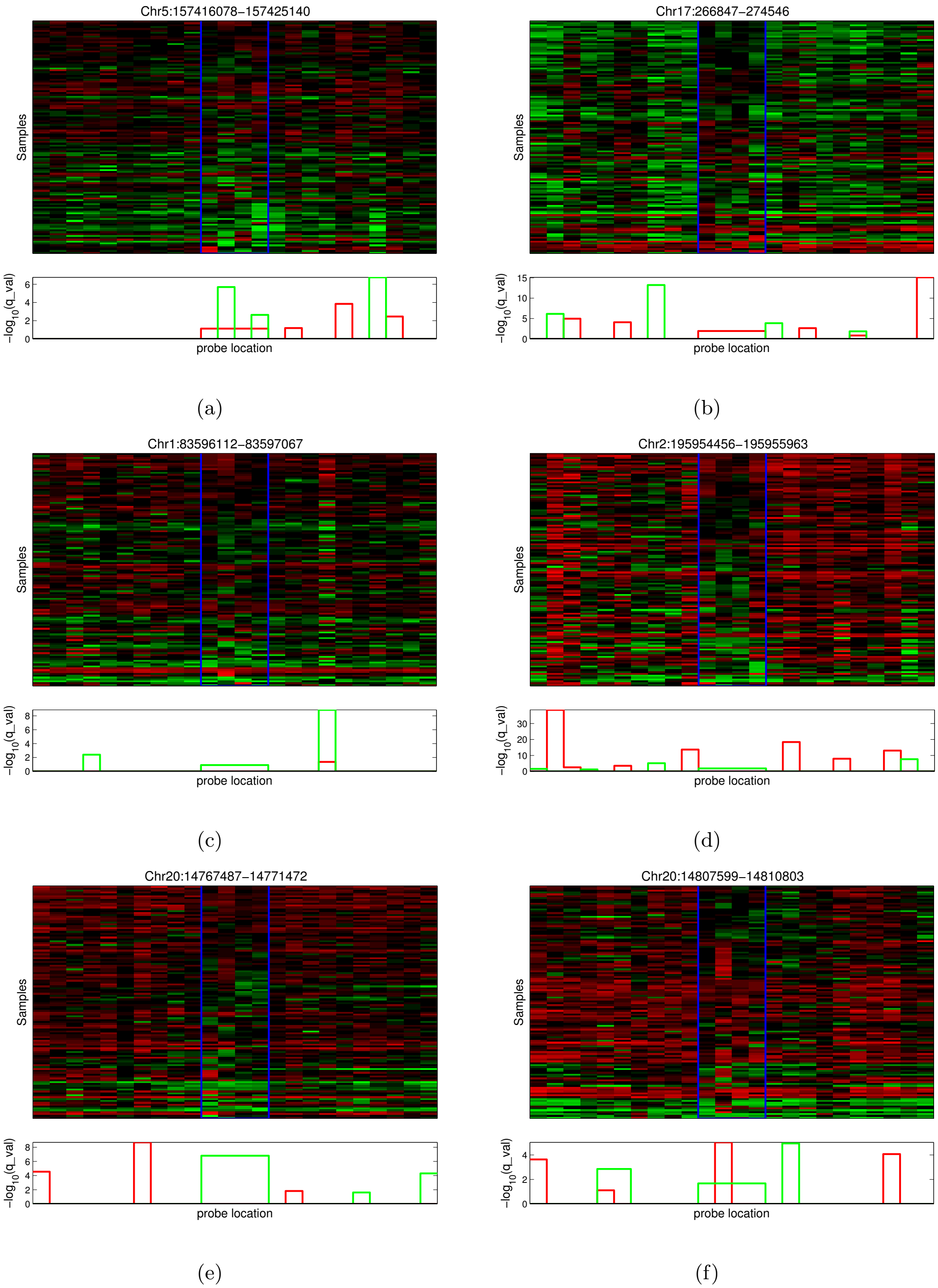

Figure S7: **Incoherent CNV patterns on ovarian cancer data.** In the heatmaps, patterns are annotated as regions between two blue vertical lines shown in original log-intensity in ovarian cancer data. The plots below the heatmaps show the *q_value* of each probe calculated by JISITC.

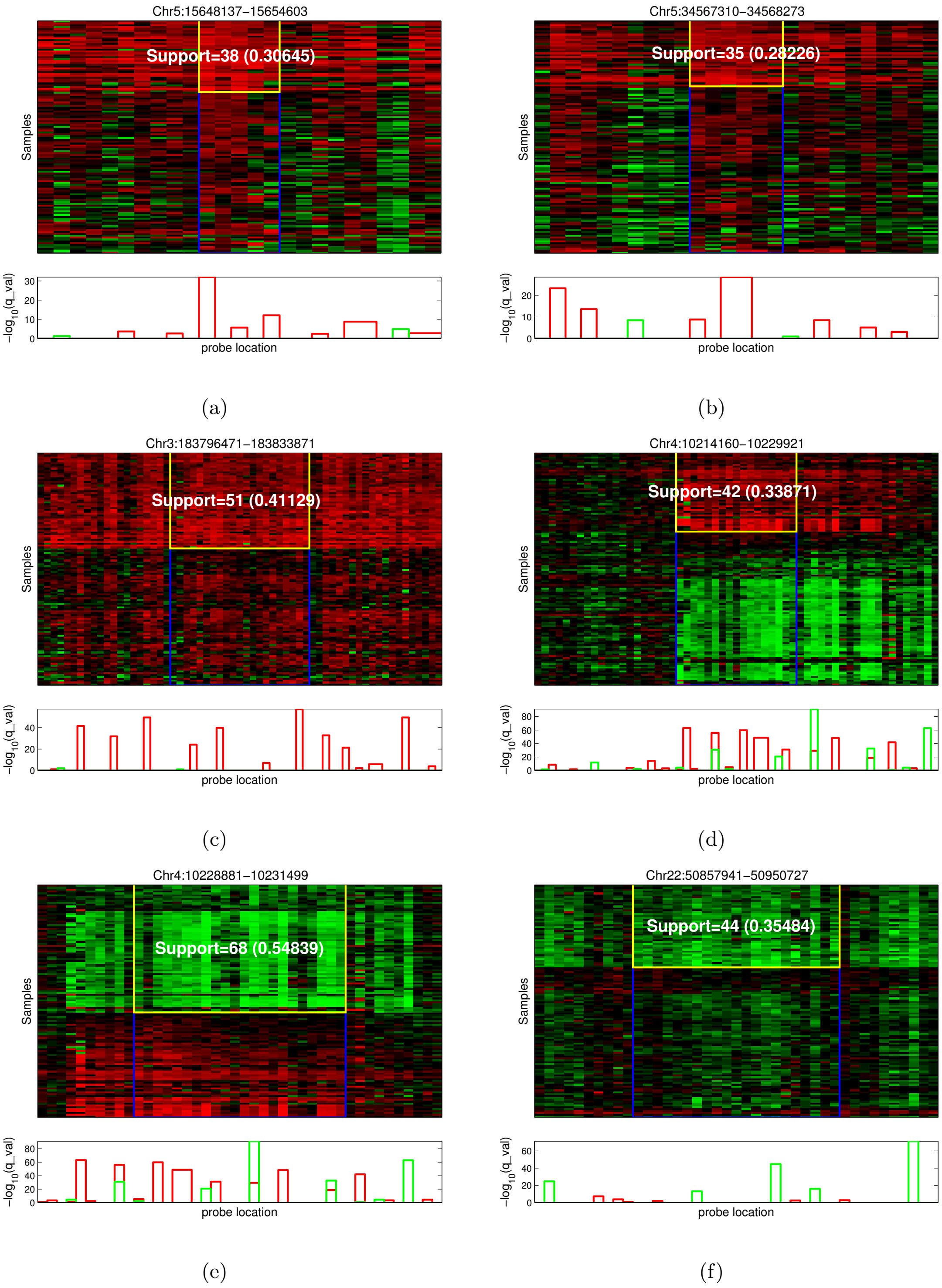

Figure S8: **Coherent patterns detected by SubPatCNV on ovarian cancer data.** The yellow rectangles mark the subset of samples that support the patterns. The plots below the heatmap show the *q_value* of each probe calculated by JISITC.

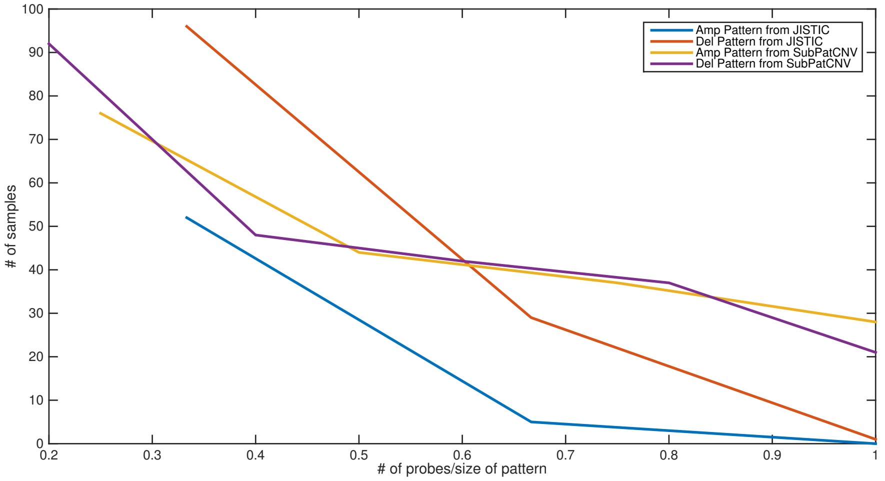

Figure S9: **Supports against the percentage of probes of the 4 patterns from the Hapmap dataset.** Each plot shows, for a certain fraction of the probes in a pattern, the maximum support associated with the probes.

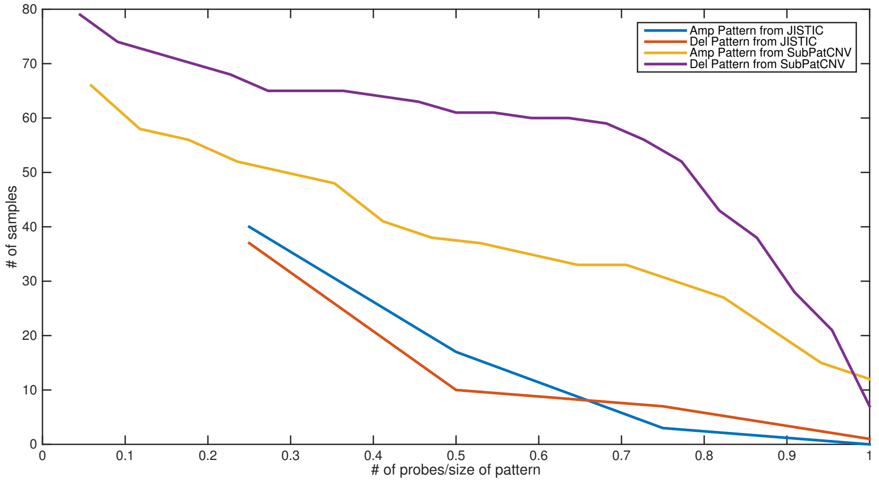

Figure S10: **Supports against the percentage of probes of the 4 patterns from the ovarian cancer dataset.** Each plot shows, for a certain fraction of the probes in a pattern, the maximum support associated with the probes.

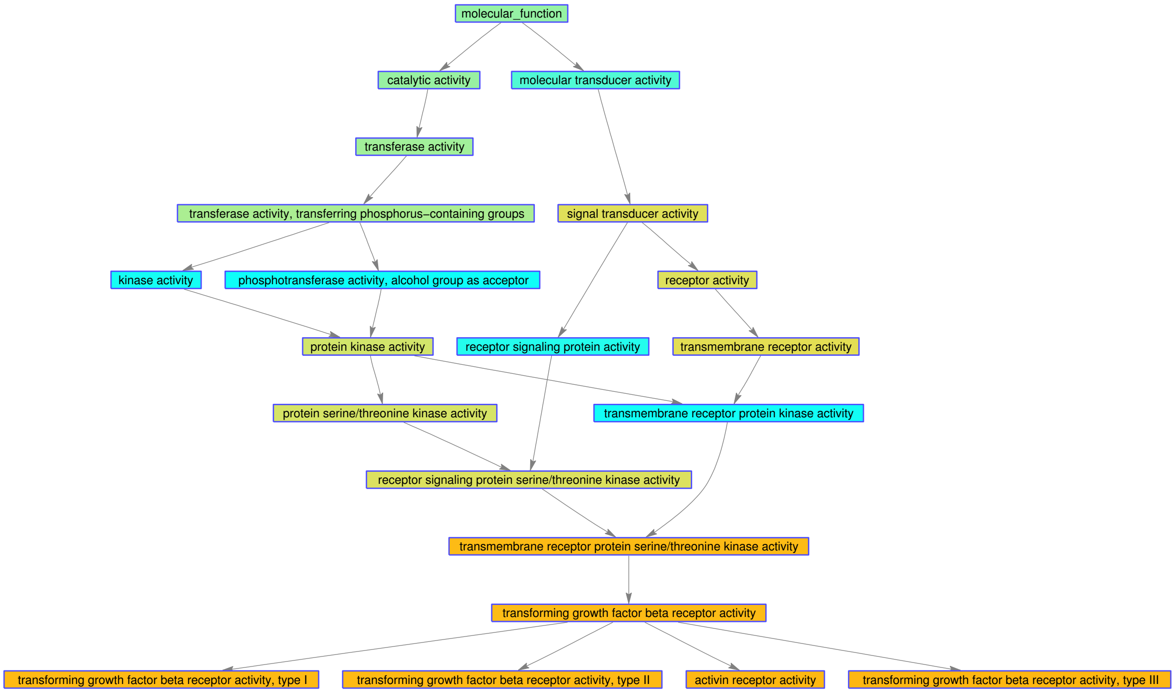

Figure S11: **Functional analysis of the ovarian cancer related genes correlated with group-specific patterns.**

support	1%	2%	5%	10%
0.05	2.2e-02	5.3e-02	7.6e-02	3.1e-01
0.10	5.0e-03	1.4e-02	2.9e-02	1.5e-01
0.20	7.7e-05	2.8e-04	1.0e-03	1.3e-02

Table S1: The density of the detected CNV patterns in the whole chromosomes under different binarization cutoff (columns) and different sample support (rows).
